# Rainstorm Disaster Risk Assessment and Influence Factors Analysis in the Yangtze River Delta, China

**DOI:** 10.3390/ijerph19159497

**Published:** 2022-08-02

**Authors:** Menghua Deng, Zhiqi Li, Feifei Tao

**Affiliations:** 1 Business School, Hohai University, Nanjing 210098, China; dengmenghua@hhu.edu.cn (M.D.); 1963710108@hhu.edu.cn (Z.L.); 2 Yangtze Institute for Conservation and Development, Hohai University, Nanjing 210098, China; 3 College of Computer and Information, Hohai University, Nanjing 210098, China

**Keywords:** rainstorm disaster, risk assessment, projection pursuit, XGBoost, Yangtze River Delta

## Abstract

Rainstorm disasters have had a serious impact on the sustainable development of society and the economy. However, due to the complexity of rainstorm disasters, it is difficult to measure the importance of each indicator. In this paper, the rainstorm disaster risk assessment framework was systematically proposed based on the disaster system theory and a system of corresponding indicators was established. Furthermore, the genetic algorithm optimized projection pursuit and XGBoost were coupled to assess the rainstorm disaster risk and to measure the relative importance of each indicator. Finally, the Yangtze River Delta was taken as the case study area. The results show that: the rainstorm disaster risk in the eastern and southeast is higher than those in the central and northwest of the Yangtze River Delta; the total precipitation from June to September and the top ten indicators contribute 9.34% and 74.20% to the rainstorm disaster risk assessment results, respectively. The results can provide references for decision makers and are helpful for the formulation of rainstorm adaptation strategies.

## 1. Introduction

Under the coupled influence of global climate change and rapid urbanization, urban rainstorms have been a frequent occurrence in recent years, bringing about serious impacts on human survival and social-economic development [1,2]. China is one of the most rainstorm disaster prone countries. There are many cities that suffer rainstorm disasters every year in China [3]. According to the Ministry of Water Resources of the People’s Republic of China, from 2010 to 2020, the total direct economic loss of rainstorm disasters was more than 410 billion dollars [4]. In 2020, rainstorm disasters in the Yangtze River Basin and Huaihe River Basin brought about direct economic losses of more than 20 billion dollars [5]. Rainstorm disaster risk assessment is the foundation of improving the rainstorm disaster defense capacity and reducing the economic loss caused by rainstorms. Rainstorm disaster risk assessment has become a hot research issue [6,7].

The occurrence of rainstorm disasters is affected by many factors. Although rainfall intensity and patterns are the direct influence of urban rainstorm disasters [8], the infrastructure [9], the underlying surface, and the disaster management level also have signification impacts on rainstorm disasters [10]. Thus, the rainstorm disaster risk assessment consists of various factors, such as natural elements, social-economic effects, the government’s intervention, and so on [11]. To assess the risk of rainstorm disasters, it is necessary to comprehensively analyze the related factors. The disaster system theory can well reflect the relationship among various factors by considering the rainstorm disaster risk assessment as a complex system [12,13]. Especially, in the background of climate change and rapid urbanization, the disaster system theory can comprehensively consider the change in inducing factors, the hazard-formulation environment, and the hazard-affected bodies. Therefore, this paper constructs a rainstorm disaster evaluation indicator system based on the disaster system theory. Based on the disaster system theory, the rainstorm disaster evaluation includes several subsystems, such as the dangerousness of hazard factors, the sensitivity of the hazard environment, the vulnerability of the hazard body, and the capability of rainstorm disaster resistance [14,15]. In the dangerousness subsystem, extreme precipitation, seasonal rainstorms, and the expansion of cities are the main factors of urban rainstorm disasters [16,17]. In the sensitivity subsystem, the elevation, the condition of the underlying surface, the water body, the cover of vegetation, the drainage network, and the historical disaster are the most important forming of disaster-pregnant environment [18,19]. In the vulnerability subsystem, the urban population, the infrastructure, and the fixed-asset investment are the vulnerability of the hazard body [20,21].

Studies on rainstorm disaster risk assessment can be classified into three aspects: historical disaster assessment methods, hydrological and hydraulic models, and index evaluation methods [3]. Historical disaster assessment methods are mainly based on mathematical-statistical methods, which require a long-term series of historical disaster data. The hydrological and hydraulic models can simulate the process of rainstorms, which can reflect the spatial characteristics of urban rainstorm disaster risk. However, they cannot comprehensively consider the impact of society and economy on rainstorm disasters. Compared with the mathematical-statistical methods and the hydrological and hydraulic models, index evaluation methods can assess rainstorm disasters with multi-dimensional characteristics and more efficiency in data utilization [22]. Scholars have adopted the analytical hierarchy process [23,24], TOPSIS (Technique for Order Preference by Similarity to an Ideal Solution) [25], coefficient of variation [26], and other methods to assess the risk of rainstorm disasters.

Based on the existing research, we know that there are numerous factors that affect the risk of rainstorm disasters. How to effectively deal with this high-dimensional problem is vital to improving the objectivity and effectiveness of the risk assessment results. Machine learning effectively deals with non-linear, complex problems with various influencing factors. It can help assess rainstorm disasters effectively and reduce the losses caused; it has been widely used in disaster assessment [27]. Based on the support vector machine (SVM) [28], multi-layer weighted principal component analysis [29], random forest [30], artificial neural network [31] and Gradient Boosting Decision Tree [32], scholars have undertaken a series of investigations on this topic. Among those machine learning algorithms, Zhao et al. verified that the effect of the ensemble machine learning model is significantly better than the individual models [33]. The XGBoost model can synthesize the benefits of each model and avoid the drawbacks, which have higher accuracy in the disaster risk assessment [34]. Meanwhile, XGBoost can predict the rainstorm disasters risk and measure the importance of each evaluation index, which is helpful to find out the key factors affecting rainstorm disaster risk. Considering that rainstorm disasters risk assessment is a complex, high-dimensional, and nonlinear problem, the projection pursuit model is effective in transferring high-dimensional data into low-dimensional data. It has been effectively used in risk evaluation fields [35,36]. Therefore, this paper establishes a rainstorm disasters risk assessment indicator system and couples projection pursuit (PP) and XGBoost to analyze the rainstorm disasters risk and to explore the influencing factors.

The Yangtze River Delta is in the plain area of the middle and lower reaches of the Yangtze River. Affected by the subtropical monsoon climate, urban rainstorm disasters occur frequently in this area, which seriously affects urban security and sustainable social-economic development. Meanwhile, with the development of regional integration in the Yangtze River Delta, the connection among cities in this area is closer than before. The occurrence of rainstorm disasters may affect transportation, communication, and other infrastructure, resulting in further economic losses. With the expansion of cities and the development of urban agglomerations, it is vital to study the spatiotemporal evolution and the influence characteristics of urban rainstorm disasters. Therefore, this paper evaluates the rainstorm disaster risk in this area.

The main contribution of the study is that: (1) the formulation of urban rainstorm disaster risk is analyzed under the climate change and urbanization background; (2) the rainstorm risk assessment index system is constructed based on the disaster system theory; (3) the rainstorm risk and the influencing factors are analyzed based on the coupled projection pursuit and XGBoost model. The study expands the research field of data analysis and provides a new solution for disaster prevention and management.

## 2. Materials and Methods

### 2.1. The Study Area and Data Source

**Figure 1:**
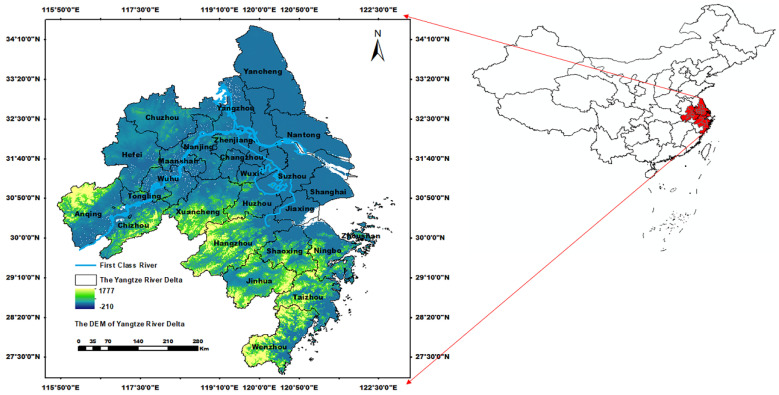
The administrative division and geographical location of the Yangtze River Basin, China.

The Yangtze River Delta (YRD) is in the plain area of the middle and lower reaches of the Yangtze River. It covers an area of more than 200 thousand km^2^ and includes 27 cities, including Shanghai, most areas of Jiangsu Province, and parts of Zhejiang and Anhui Province (Figure 1) [37]. The Yangtze River Delta is one of the most economically developed regions in China. The population was stated to be more than 200 million residents at the end of 2019 [38]. However, affected by the subtropical monsoon, there are many typhoons and rainstorms in the summer, which are characterized by the high intensity and concentrated range. More than 60% of annual precipitation is concentrated from June to September, resulting in frequent basin or urban rainstorm disasters [13]. In recent years, summer precipitation in the Yangtze River Delta has shown an increasing trend [39]. Meanwhile, the main surface cover of the Yangtze River Delta has transferred from cropland to built-in land. Shanghai, especially, has experienced a fast increase in the proportion of built-in land, from 23% in 2000 to 39% in 2020 [38]. Along with the rapid economic development and urbanization, the affected population, deaths, crops, and direct economic loss by rainstorms disasters are increasing, and the risk of rainstorm disasters in Jiangsu, Anhui, and Zhejiang has an upward tendency [40,41]. Thus, it is important to assess the risk of rainstorm disasters to ensure urban water security, and to realize the integrated and high-quality development of the Yangtze River Delta.

According to the disaster system theory and the existing research, this paper selected indicators from four aspects: dangerousness, sensitivity, vulnerability, and capability [12,13]. The precipitation data are from the China Meteorological Administration and the urbanization rate and the sensitivity indicators data are from the “China Urban Construction Statistical Yearbook”. The unit area GDP (Gross Domestic Product) and population density are from the Resource and Environmental Science Data Center of the Chinese Academy of Sciences. Other data are from the Statistical Yearbook of each province and city.

### 2.2. The Framework of Rainstorm Disaster Risk Assessment

**Figure 2:**
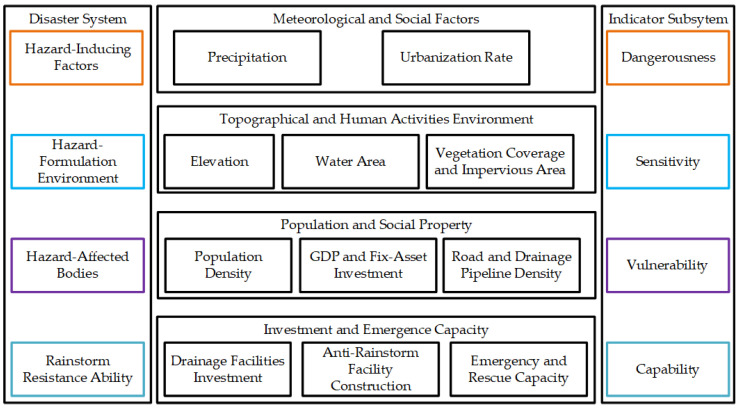
The framework of rainstorm disaster risk assessment based on disaster system theory.

Rainstorms are the extreme manifestation of the urban water circulation system. They are a natural disaster, in which the accumulated water cannot drain away timely and effectively. The occurrence of rainstorm disasters is due to the interaction of the natural environment and human society. From the perspective of the natural environment, global warming has changed the climate and has affected the urban hydrology environment, increasing the frequency and intensity of extreme rainfall. From the perspective of human society, human activities have affected the urban water cycle in all aspects. For example, urbanization and the increase in the impervious areas of the urban surface have affected the urban climate and the runoff of urban surfaces. Generally, the formulation of rainstorm disasters is the comprehensive result of the inducing factors, the hazard-formulation environment, and the hazard-affected bodies. Meanwhile, if we can deepen our understanding of rainstorms, humans can undertake a series of measures to avoid or reduce the losses caused by rainstorms. Thus, from the perspective of disaster system theory [12], the occurrence of rainstorm disasters is the interaction among the dangerousness of hazard-inducing factors, the sensitivity of hazard-formulation environment, the vulnerability of hazard-affected bodies, and the capability of rainstorm disaster resistance (Figure 2).

As is shown in Figure 2, precipitation, especially a large amount of precipitation in a short time, is the main factor that leads urban rainstorm disasters. According to historical statistics, the Yangtze River Delta is prone to rainstorm disasters from June to September every year. Meanwhile, with the acceleration of urbanization, the heat island effect of urbanization increases the intensity of convective precipitation and the frequency of thunderstorms in the summer. Climate change and urbanization have become the most inducing factors of urban rainstorm disasters. Therefore, we chose the maximum daily precipitation (24-H-P), the maximum monthly precipitation (M-D-P), the total precipitation from June to September (JS-T-P) and the urbanization rate (UR) as the dangerousness indicators. Generally, the higher value of the dangerousness indicators, the higher the rainstorm risks will be. The hazard-formation environment is the geographical and topographic status of the urban environment. The elevation and distribution of the water system and the coverage of vegetation are closely related to the risk of rainstorm disasters. Meanwhile, with the higher density of vegetation, there will be more infiltration and the formation of ponding will be slower. This is the same per capita public green space. Meanwhile, the water area ratio represents the distribution of the urban water network. Generally, the higher density of the water network, the higher probability of rainfall accumulation, and the higher risk of rainstorm disasters. With the rapid development of urbanization, the urban road and buildings are increasing, resulting in the expansion of the impervious areas and the hardening of the underlying surfaces. The increase in urban impervious areas has changed the urban natural water circulation system and declined regional water storage and urban drainage capacity, resulting in the increase in surface flow and the risk of rainstorm disasters. The per capita public green space area (PGS) is closely related to the urbanization rate, the use of land, and the population density [42]. Generally, the higher the PGS, the lower the rainstorm risk. Therefore, we chose elevation (Elev), vegetation coverage ratio (VCR), PGS, water area ratio (WAR), and the urban impervious area ratio (UIAR) as the sensitivity indicators. Generally, the higher value of Elev, VCR, and PGS, the lower the risk level will be. Additionally, the higher value of WAR and UIAR, the higher the risk will be. The hazard-affected body is a complex system, including economic factors, environmental factors, and social factors. The vulnerability is used to measure the loss possibility of the hazard body when facing rainstorm disasters. Generally, the economic loss caused by the same level of a rainstorm in different regions may vary greatly. The loss in densely populated and economically developed areas may suffer much more than those in sparsely populated and less developed areas. The urban drainage pipe network behind the rapid development of urbanization, resulting in the high vulnerability of hazard bodies. Therefore, the population density (PD), unit area GDP (UA-GDP), unit area fixed-asset investment (UA-FAI), the road density in the built-up district (RD-BUD), and the drainage pipeline density in the built-up district (DFD-BUD) are selected to measure the vulnerability of the hazard body. Generally, the higher value of PD, UA-GDP, UA-FAI, and RD-BUD, the higher the rainstorm risk level. However, the higher value of DFD-BUD, the lower the rainstorm risk level will be.

The capability of rainstorm disasters resistance indicates the ability to avoid and recover from disasters. It is closely related to the investment in the drainage facilities, and the government’s disaster prevention and reduction investment. The rescue and response capability of the public government and people can effectively reduce the losses of rainstorm disasters. Therefore, we selected the unit area drainage facilities investment (UA-DFI), anti-rainstorm disaster facility construction level (ARFCL), government emergency and rescue capacity (GERC) and the public disaster emergency response capacity (PDERC) to measure the ability of rainstorm disaster prevention and reduction. Generally, the higher value of the capability indicators, the lower the rainstorm disaster risk will be.

Based on the above analysis and previous studies [8,9,10,11,12,13], the indicator system is constructed in Table 1.

### 2.3. Rainstorm Disaster Risk Assessment Based on GAPP-XGBoost

In this paper, the GAPP and XGBoost are coupled to assess the rainstorm disaster risk and to measure the importance of each indicator. Firstly, the best projection direction and the optimized projection values are calculated based on the GAPP algorithm; then, the risk level is calculated according to the projection values; finally, the contribution of each index to the rainstorm disaster risk is measured based on the XGBoost.

#### 2.3.1. Genetic Algorithm Optimized Projection Pursuit (GAPP)

Projection pursuit (PP) projects the high-dimensional data into the low-dimensional subspace to reflect the structure or characteristics of the original high-dimensional data [43]. It can effectively overcome the problem of “Curse of Dimensionality” and achieve the purpose of studying and analyzing the nonlinear and non-normal data. Suppose the number of samples is m, and the number of indicators is n, the evaluation sample set is $X=\left\{x_{ij}|i=1,2,\cdots ,m;\;j=1,2,\cdots ,n)\right\}$. The calculation steps are as follows [44]:

- Normalize the sample set

As different indicators have different scales and units, to facilitate the comparisons among various indicators, it is necessary to normalize each indicator range from zero to one. In this paper, the min-max standardization method is used to normalize the sample set. The formulas are expressed as follows:

(1) $$x_{ij}^{*}=\left\{ \begin{array}{l} \frac{x_{ij}^{}-\min(x_{j}^{})}{\max(x_{j}^{})-\min(x_{j}^{})},\;\mathrm{for}\;\mathrm{the}\;\mathrm{positive}\;\mathrm{indexes}\\ \frac{\max(x_{j}^{})-x_{ij}^{}}{\max(x_{j}^{})-\min(x_{j}^{})},\;\mathrm{for}\;\mathrm{the}\;\mathrm{negative}\;\mathrm{indexes} \end{array}\right.$$

where $\max(x_{j}^{})$ and $\min(x_{j}^{})$ are the maximum and minimum value of the jth index, respectively. $x_{ij}^{*}$ is the normalized value of the sample set.

- Dimensionality reduction

To measure the comprehensive level of each sample, the projection pursuit (PP) algorithm projected the n-dimensional sample data into a one-dimensional projection value. Suppose there is a unit length vector $a=\left\{a_{1},a_{2},\cdots ,a_{n}\right\}$, then the projection values can be calculated as follows:

(2) $$z\left(i\right)=\sum_{j=1}^{n}a_{j}x_{ij}^{*},\;i=1,2,\cdots ,m$$

where z(i) is the projection value of ith sample, $a_{j}$ is the projection direction value of the jth index.

- Construct the projection index function

The projection index function can be calculated as follows:

(3) $$Q\left(a\right)=S_{z}D_{z}$$

where $S_{z}$ and $D_{z}$ are the standard deviation and local density of z(i), respectively. They can be calculated as follows:

(4) $$S_{z}=\sqrt{\frac{\sum_{i=1}^{n}{\left(z\left(i\right)-E\left(z\right)\right)}^{2}}{n-1}}$$

(5) $$D_{z}=\sum_{i=1}^{n}\sum_{j=1}^{n}\left(R-r_{ij}\right)\cdot u\left(R-r_{ij}\right)$$

where $r_{ij}$ is the distance among samples, E(z) is the mean value of z(i), R is the window radius of $D_{z}$, and u(t) is the unit step function. When t≥0, u(t)=1; when t<0, u(t)=0.

- Optimize projection index function

Regarding the projection index function as the objective function, the dimension reduction transformation rule as the constraint condition, then we can obtain the following optimization function:

(6) $$Max\;Q\left(a\right)=S_{z}D_{z},\;s.t.\;\sum_{j=1}^{n}a_{j}^{2}=1$$

The above function is a complex nonlinear constraint problem. The Genetic Algorithm (GA) is a global optimization search method based on the theory of genetic mechanisms. By transforming the resolved problem into the selection, crossover, and mutation of chromosome genes, the GA method can well resolve the complex combinatorial optimization problems. It has been widely used in multi-objective optimization [19], pattern recognition [45], and social-science research. Therefore, in this study, GA was introduced to optimize the PP algorithm.

- Classify and evaluate the sample set

Based on the best projection direction and Equation (2), we can obtain the optimized projection value. According to the quantitative and the optimized projection value, the function concerning the risk level and projection value can be established; that is, the evaluation model.

(7) RL=f(z)

where *RL* is the risk level, and *z* is the optimized projection value. Using the above function, the risk level can be obtained, and the risk map can be drawn, which can provide a decision making reference for disaster prevention and emergency.

#### 2.3.2. Extreme Gradient Boosting (XGBoost)

XGBoost is an ensemble machine learning algorithm based on the advanced implementation of the gradient boosting algorithm [46]. The algorithm utilizes the CART (Classification and Regression Tree) as the base classifier and runs cross-validation at each iteration, which makes it easy to obtain the optimum number of boosting iterations. The algorithm can well handle irregular data and missing data, and the performance is faster and better than other decision tree algorithms [34]. Meanwhile, the algorithm is highly flexible so that the user can define objective functions and evaluation criteria to improve accuracy.

Suppose the number of samples is m, and the number of indicators is n, the evaluation sample set is $X=\left\{x_{ij}|i=1,2,\cdots ,m;\;j=1,2,\cdots ,n)\right\}$, and the number of decision trees is *K*. Then, the predicted value of *i*th sample is jointly decided by the *K* trees can be expressed as below:

(8) $${\overset{⌢}{y}}_{i}=\sum_{k=1}^{K}f_{k}(x_{i}),f_{k}\in \;F$$

where ${\overset{⌢}{y}}_{i}$ is the predicted value, $f_{k}$ is the *k*th tree, $f_{k}(x_{i})$ is the score of the *i*th sample in the *k*th tree. F is the all possible of decision trees.

The objective function of the XGBoost algorithm is composed with the loss function and the regularization term, which can be expressed as follows:

(9) $$L(\phi )=l(\phi )+\Omega (\phi )=\sum_{i=1}^{m}l({\hat{y}}_{i},y_{i})+\sum_{k=1}^{K}\Omega (f_{k})$$

(10) $$\Omega (f)=\gamma T+\frac{1}{2}\lambda {\left\|\omega \right\|}^{2}$$

where, l(ϕ) is the loss function, which can measure the training error; the Ω(ϕ) is the regularization term, used to penalize the complexity; *T* is the number of leaf nodes in each decision tree; γ is the complexity of each leaf nodes; λ is used for scaling the penalty; ω is the collection scores of leaf nodes scores of each decision tree.

Assume ${\hat{y}}_{i}^{t}$ is the predication value of the *i*th sample at the *t*th iteration, then the objective function is as follows:

(11) $$\Gamma^{\left(t\right)}=\sum_{i=1}^{m}l[({\hat{y}}_{i}^{t-1},y_{i})+f_{t}(x_{i})]+\Omega (f_{t})$$

Taylor Formula is used to express Equation (11), the optimized objective function is as follows:

(12) $$\Gamma^{t}≅\sum_{i=1}^{m}\left[l(y_{i},{\hat{y}}_{i}^{t-1})+g_{i}f_{t}(x_{i})+\frac{1}{2}h_{i}f_{t}^{2}(x_{i}\right)]+\Omega (f_{t})$$

where $g_{i}=\partial_{\overset{⌢}{y}(t-1)}l(y_{i},{\overset{⌢}{y}}^{\left(t-1\right)})\;and\;h_{i}=\partial_{\overset{⌢}{y}(t-1)}^{2}l(y_{i},{\overset{⌢}{y}}^{\left(t-1\right)})$. We transform the iteration of the tree model into the iteration of the leaf nodes, then we can obtain the scores of the optimal leaf nodes. Bring the values to the objective function, we can obtain the optimal value as the follows:

(13) $${\tilde{\Gamma }}^{t}(q)=-\frac{1}{2}\sum_{j=1}^{T}\frac{{\left(\sum_{i\in I_{j}}g_{i}\right)}^{2}}{\sum_{i\in I_{j}}h_{i}+\lambda }+\gamma T$$

The above function is the score function, which can measure the structural quality of the qth tree. To make XGBoost run more quickly and obtain higher accuracy, we need to tune the parameters to improve speed and accuracy of the model. In this paper, the number of trees with different learning rates is determined; then the grid search method is used to explore the optimal values of each parameter; finally, the accuracy of the algorithm and the evaluation results are obtained.

#### 2.3.3. Rainstorm Disaster Risk Assessment Based on GAPP-XGBoost

**Figure 3:**
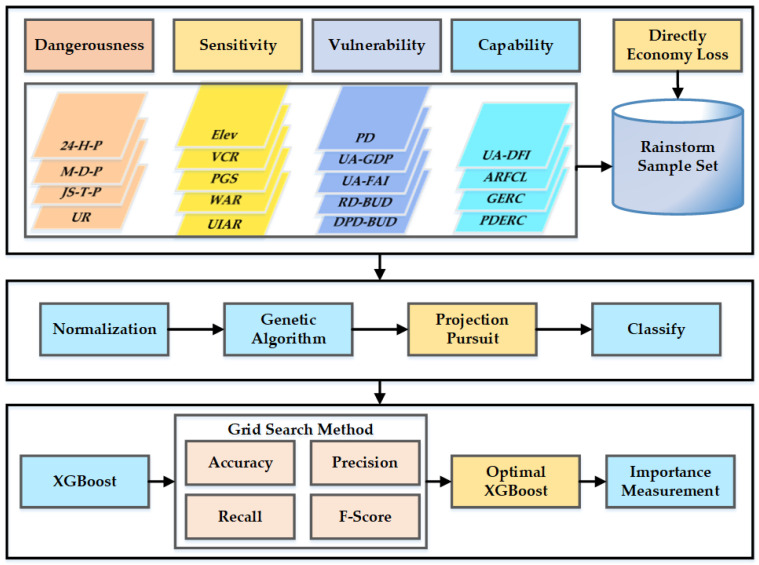
Rainstorm disaster risk assessment and influencing factors analysis based on GAPP-XGBoost.

In this paper, the GAPP and XGBoost model are coupled to assess the rainstorm disaster risk and to analyze the influencing factors. The steps of the whole study are shown in Figure 3.

As is shown in Figure 3, firstly, the precipitation, population, GDP, and other indicators of the 27 cities in the Yangtze River Delta from 2011 to 2019 were collected. Then, the collected data were normalized, and projection values were obtained based on the genetic algorithm optimized projection pursuit model. Thirdly, the rainstorm disaster risk level was calculated based on the projection values. Finally, the importance of each indicator of rainstorm disaster risk level was measured based on XGBoost.

## 3. Results

**Figure 4:**
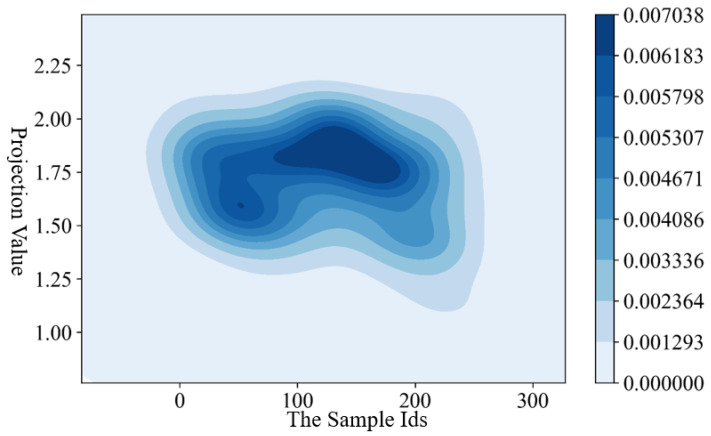
The projection values of 27 cities from 2011 to 2019.

The GAPP and XGBoost model are used to assess the rainstorm disaster risk from a spatial and temporal perspective. Firstly, the samples are normalized; then the best projection direction and the optimized projection values are calculated based on the GAPP algorithm; finally, the risk level is calculated according to the projection values. In the GAPP model, to optimize the objective function, we set the number of breeding algebra to 50, the population number of each generation is 30, the crossover probability is 0.6, and the mutation probability is 0.01. Then, the values of the optimized projection values are shown in Figure 4.

**Figure 5:**
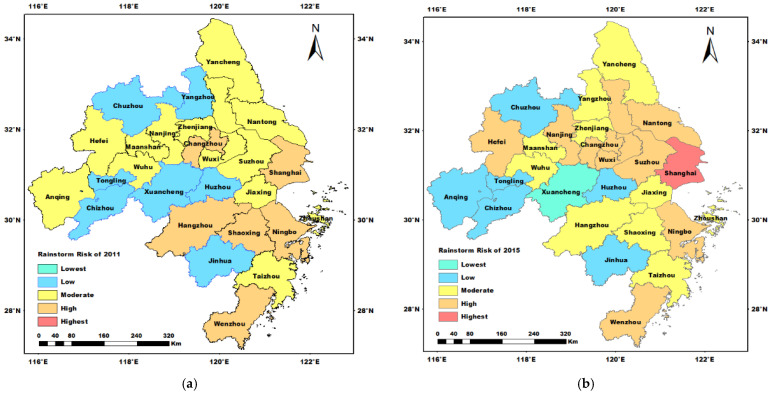

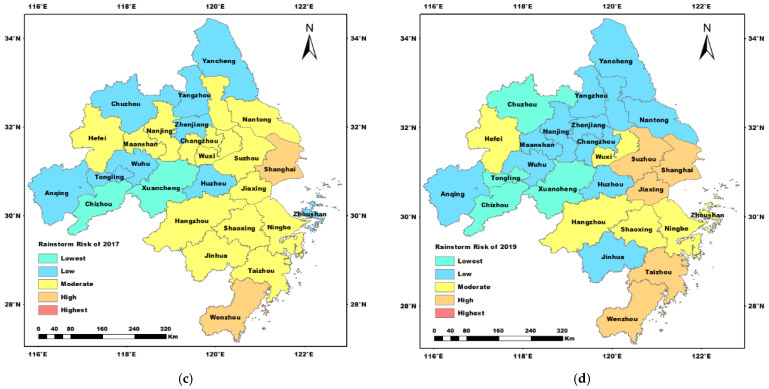
The spatio-temporal distribution of rainstorm disaster risk level of 27 cities from 2011 to 2019 (**a**) the rainstorm disaster risk of 2011; (**b**) the rainstorm disaster risk of 2015; (**c**) the rainstorm disaster risk of 2017; (**d**) the rainstorm disaster risk of 2019.

As shown in Figure 4, the projection values are relatively clustered. Most of the values are distributed between 1.4 and 2.0. Affected by the special geographical location and climate conditions, the Yangtze River Delta is prone to rainstorm disasters. Recently, the government has taken a series of engineer and non-engineer measures to prevent rainstorm disasters, which has had a positive effect on the reduction in rainstorm loss [7,47]. Based on the Z-scores method [48] and the classification standard of rainstorm disasters [13], the rainstorm disaster risk was divided into five levels. Then, the relationship between projection values and the rainstorm disaster risk level was established. The risk zoning maps are shown in Figure 5.

Due to the space constraints, and for a better illustration, we only provided the rainstorm disaster risk in the years 2011, 2015, 2017, and 2019. As is shown in Figure 5, the rainstorm disaster risk levels in the eastern and southern regions are higher than that in the western and northern regions, respectively. The lower rainstorm disaster risk cities are mainly in the central and northwest of the Yangtze River Delta. For example, due to the low frequency and less amount of precipitation, the rainstorm disaster risk of Huzhou is relatively lower than the risk of other cities. The cities in the northwest have lower rainstorm disaster risk, mainly due to the low density of the river network and population, the higher elevation, and the relatively less developed economy. The eastern coastal and southeastern areas with concentrated populations and developed economies have higher rainstorm disaster risk, such as Shanghai, Hangzhou, Ningbo, etc. Meanwhile, due to the high level of social and economic development, the cities in the Yangtze River Delta generally have good disaster prevention and reduction ability. Generally, the areas with high economy developed have higher disaster prevention and reduction ability. As is shown in Figure 5, the rainstorm risk level increased in Shanghai during 2011–2015 and decreased in the period 2017–2019. The geological and climatic conditions mean that Shanghai is prone to suffer rainstorm disasters. From 2011 to 2017, the rainstorm disaster risk demonstrates an upward trend [49]. The hard and soft protection strategies can reduce the rainstorm risk effectively [47]. Meanwhile, due to the geographical location and climatic conditions, the rainstorm risk level of Wenzhou is high throughout the studied periods. According to the previous study, Wenzhou is located on the southeast coast of China, which is prone to typhoons and faces the highest risk of rainstorm disasters [50]. To further explain and discuss the spatiotemporal distribution of rainstorm risk levels, the percentage of rainstorm risk classes during the five reference years is shown in Table 2.

**Figure 6:**
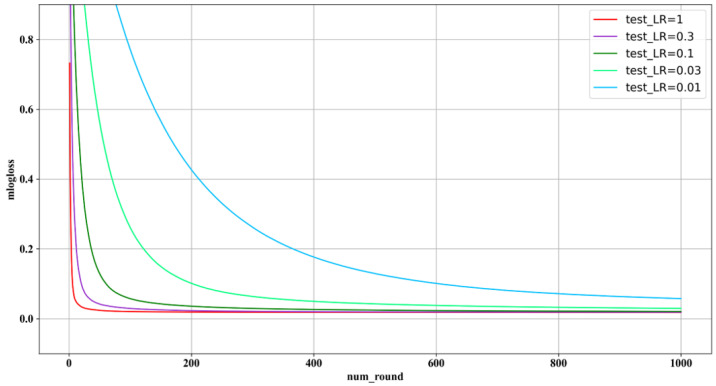
The number of estimates trees under different learning rates.

As is shown in Table 2, from 2011 to 2019, the lowest and low level of rainstorm risk demonstrates an upward trend, while the moderate and high levels of rainstorm rise first and then decrease. According to historical data, the precipitation from June to September (JS-T-P) of most cities in 2019 is lower than that in 2017. However, with the rapid development of urbanization and climate change, the urban impervious area and population density are increasing. To explore the influencing factors of rainstorm disasters, we chose the XGBoost model to verify the evaluation results and measure the contribution of each index to the rainstorm disaster risk. To improve the accuracy of the XGBoost model, there are several parameters that need to be tuned: for example, the number of estimates of each tree, the max depth of each tree, the learning rate, and so on. Generally, as the number of estimated trees increases, the stability of the model is better. However, the improvement is limited when the number of trees increases to a certain value. The optimal number of estimated trees under different learning rates is shown in Figure 6.

**Figure 7:**
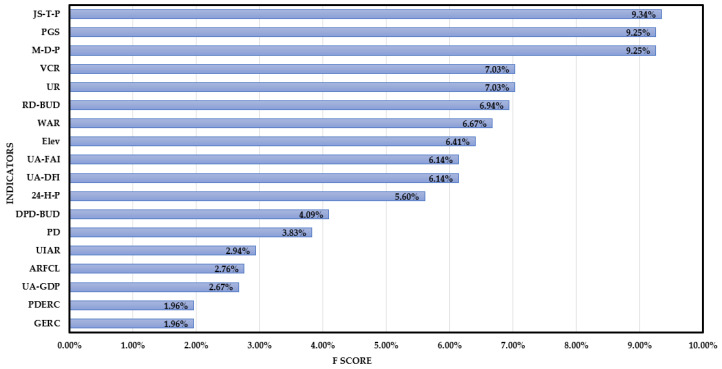
The importance of each indicator to rainstorm disaster risk.

As is shown in Figure 6, when the learning rate is equal to 0.3 or 1, the best-estimated trees are no more than 100. Additionally, when the learning rate is equal to 0.03 or 0.01, the best-estimated trees are less than 500. The optimal values of other parameters were obtained through the grid search method. We obtained the optimal parameter’s value set in which the classifier is softmax, the learning rate is 0.3, the max depth of each tree is 6. Then, the importance of each indicator was measured based on the XGBoost model. The importance of the indicators is shown in Figure 7.

As is shown in Figure 7, the importance of each indicator is significantly different. The bigger the values, the more important the index is. The value of the total precipitation from June to September (JS-T-P), the maximum monthly precipitation (M-D-P), and per capita public green space (PGS) account for 9.34%, 9.25%, and 9.25%, respectively, which means they are the significant factors in the whole index system. With the development of the economy, and the progress of science and technology, the anti-rainstorm disaster infrastructure is strengthening, and the rainstorm disaster control measures are improving, effectively reducing the risk level, and avoiding economic loss and casualties. However, due to the unique geographical location and climatic conditions of the Yangtze River Delta, most of the rainstorm disasters occur from June to September each year. In the rainy season, a large amount of precipitation rises the river water level, affecting the surface runoff and the effect of the drainage pipe network, resulting in the high risk of rainstorm disasters. The green space is conducive to regulating and storing rainwater, reducing surface runoff and the risk of rainstorm disasters. The followed indicators are urbanization rate (UR), vegetation coverage ratio (VCR), the road density in the built-up district (RD-BUD), the water area ratio (WAR), Elevation (Elev), unit area fixed-asset investment (UA-FAI), and unit area drainage facilities investment (UA-DFI), accounting for 6.14% of the total importance. The less coverage of vegetation, the easier it is to form the surface runoff and peak, which increases the risk level of rainstorm disasters. Water area ratio is also an important factor that influences rainstorm disaster risk. Affected by the rapid expansion of urbanization, the original river network is damaged; the regulation and storage capacity cannot adjust to the urban development, resulting in increased pressure on rainstorm disaster control and the probability of rainstorm disasters.

## 4. Discussion

Urban rainstorm disasters have affected the healthy development of society and the economy [7]. This paper chose the Yangtze River Delta as an example to assess the rainstorm disaster risk and to identify the influence indicators to provide effective guidance on rainstorm disaster control. The results show that the frequency of rainstorm disasters in the eastern and southeast of the Yangtze River Dela is higher than that in the central and northwest. Due to climate change, most coastal cities are sensitive to rainstorm risks, meaning that the rainstorm risk in eastern coastal cities is higher than in the central cities [49,50]. Furthermore, due to the rapid urbanization and the changing environment, from 2011 to 2017, the rainstorm disaster risk demonstrates an upward trend, which is consistent with the results of Sun et al. [51]. Thus, it is necessary to further improve the anti-rainstorm disaster capability and the emergency management mechanisms [47]. The influence analysis shows that the three most important indicators are the total precipitation from June to September (JS-T-P), the per capita public green space (PGS), and the maximum monthly precipitation (M-D-P). Affected by geographical location, climatic conditions, and typhoons, the Yangtze River Delta is prone to rainstorm disasters from June to September [9]. According to the relative importance of the top three indicators, the most effective to reduce the risk of rainstorm disaster is to improve the early warning ability of urban rainstorm disaster risk, and the accuracy of the rainstorm forecast. Meanwhile, increasing the green area is also effective in decreasing runoff and delaying the rainstorm peak. The emergency and rescue capacity of the government cannot change significantly in the short term, meaning that GERC is less important than other indicators [7]. Due to the rapid development of urbanization, the construction of drainage pipelines lags behind urban development, restricting its role in the relief of urban rainstorm disasters. Although the importance of drainage pipeline density in the built-up district (DPD-BUD) is lower than expected, the government should take measures to improve the drainage capacity.

Considering the indicators, whether easy to control or not, the government can take corresponding countermeasures. Based on the above influence analysis results, the total precipitation from June to September (JS-T-P) is one of the most important indicators; thus, the government can improve the rainstorm warning and emergency management mechanism. Meanwhile, it is important to make information on disaster prevention publicly available and educate people through social media: for example, radio, television, newspapers, the mobile internet, WeChat, and take other measures to improve the public’s consciousness and their self-help abilities in regard to rainstorm disasters. Due to the rapid urbanization and the increasing impervious area, cities should improve the drainage system and make it adapt to the development of urbanization and, finally, develop ecological resilient cities. These rainstorm-resilient cities will be able to combine urban construction with ecological community construction, transform the urban community with the concept of human natural ecological harmony and realize ecological water storage and drainage to enhance the ecological resilience of urban waterlogging prevention and control.

## 5. Conclusions

Under the background of climate change and rapid urbanization, this paper constructed an urban rainstorm disaster risk assessment framework and established corresponding indicator systems. Taking the Yangtze River Delta as a case study area, the risk levels were assessed, and the relative importance of each indicator was analyzed based on the genetic algorithm optimized projection pursuit and the XGBoost model. The results show that rainstorm disaster risk in the eastern and southeast is higher than that in the central and northwest of the Yangtze River Delta. The influence factors analysis results show that JS-T-P, M-D-P, PGS, UR, VCR, RD-BUD, WAR, Elev, UA-FAI, and UA-DFI are more important than other indicators.

This paper established a rainstorm disaster risk assessment index system based on the disaster system theory, which provides a solution for scientifically and objectively constructing the index system. Meanwhile, based on projection pursuit and XGBoost, the rainstorm disaster risk and the influence factors were analyzed in a data-driven way, expanding the research field of data analysis and providing a new solution for the exploration of the disaster mechanism. The indicator system and the research can provide references for other disaster risk assessments and the evaluation results can provide decision-making references for formulating rainstorm disaster risk prevention and control measures. However, this study also has some limitations and weaknesses. If the study period is longer, the statistical meaning may be better. Meanwhile, the indicators require more in-depth exploration. In future studies, the improved XGBoost, as well as other intelligent algorithms, can be explored to improve the assessment results of rainstorm disaster risk.

## Figures and Tables

**Table 1:** The indicator system of rainstorm disaster risk assessment.

	Abbreviation	Name
Dangerousness	24-H-P	maximum daily precipitation
	M-D-P	the maximum monthly precipitation
	JS-T-P	the total precipitation from June to September
	UR	urbanization rate
Sensitivity	Elev	elevation
	VCR	vegetation coverage ratio
	PGS	per capita public green space
	WAR	water area ratio
	UIAR	the urban impervious area ratio
Vulnerability	PD	population density
	UA-GDP	unit area GDP
	UA-FAI	unit area fixed-asset investment
	RD-BUD	the road density in the built-up district
	DPD-BUD	the drainage pipeline density in the built-up district
Capability	UA-DFI	unit area drainage facilities investment
	ARFCL	anti-rainstorm facility construction level
	GERC	government emergency and rescue capacity
	PDERC	the public disaster emergency response capacity

**Table 2:** Each risk level percentage during 2011, 2013, 2015, 2017, and 2019.

Risk Level	Lowest	Low	Moderate	High	Highest
2011	0%	25.93%	51.85%	22.22%	0%
2013	3.70%	40.74%	40.74%	11.11%	3.70%
2015	3.70%	22.22%	37.04%	33.33%	3.70%
2017	7.41%	33.33%	51.85%	7.41%	0%
2019	14.81%	44.44%	22.22%	18.52%	0%

## Data Availability

Not applicable.
